# The potential and value of epidemiology in curbing non-communicable diseases

**DOI:** 10.1017/gheg.2016.10

**Published:** 2016-09-09

**Authors:** A. Patel, R. Webster

**Affiliations:** The George Institute for Global Health, University of Sydney, Australia

**Keywords:** Epidemiology, global health, non-communicable diseases, public health

## Abstract

Non-communicable diseases (NCDs) have reached pandemic levels globally and pose a major threat to social and economic development worldwide. The discipline of epidemiology has done much to bring this issue to the forefront of global health. Epidemiological approaches have broadened our understanding of the impact of NCDs in widening socioeconomic disparities. Over a number of decades, this discipline has also contributed to the development of many preventive measures and treatments of known efficacy and safety. However, epidemiology also has a critical role to play in better translating these discoveries into practice, through the new science of implementation. As we strive to achieve the “25 by 25” goal of a 25% reduction in premature mortality from common NCDs by 2025, the discipline of epidemiology will need to continuously evolve to remain an essential tool for public health action.

In November 2009, the Economist published an article accusing the World Health Organization (WHO) of ‘mission creep’ and ‘nannying’ in response to their efforts to raise global attention to the perils associated with obesity, smoking and other ‘non-infectious ailments’ [1]. Branding such conditions as ‘lifestyle diseases’ occurring as a consequence of personal choice, the magazine cajoled multilateral agencies to avoid focusing on conditions associated with affluence and instead concentrate efforts on infectious diseases affecting developing countries. Fast forward to September 2011 and the UN High Level Meeting on Non-communicable Diseases (NCDs) was held in New York – only the second time the UN has convened such a meeting relating to a global health issue, the first being in response to HIV/AIDS in 2001. The ensuing Political Declaration recognised the global threat that NCDs pose, identifying these conditions as one of the major challenges facing social and economic development globally and an important contributor to rising inequalities both within and between countries [2]. Subsequently at the 65th World Health Assembly in 2012, WHO member states made a commitment to reduce premature deaths from a group of specified NCDs [cardiovascular diseases (CVD), diabetes, cancer and chronic respiratory diseases] by 25% by the year 2025 (‘25 × 25’ goal). This was followed by adoption of a global monitoring framework of nine voluntary global NCD targets that focuses on risk factors such as tobacco and alcohol use, physical inactivity, high salt intake, high blood pressure, diabetes and obesity, as well as the availability of basic technologies and medicines for the prevention and treatment of major NCDs [3].

This wider acceptance of NCDs as an important global health threat owes much to the discipline of epidemiology – that branch of medical science dealing with the incidence, distribution and opportunities for control of diseases and their determinants. Particularly noteworthy is the pivotal work of the Global Burden of Disease (GBD) consortium. A landmark publication in 2001 provided a comprehensive global and regional assessment of morbidity and mortality associated with diseases and their risk factors, using the new and subsequently widely-used metric of disability-adjusted life years (DALYs) [4]. Particularly important were the comparisons made between estimates of disease burden in 1990 and 2001, which highlighted the rapid epidemiological transition occurring in most low and middle income countries (LMIC) towards NCDs becoming leading causes of morbidity and premature mortality. Despite limitations reflecting available primary data (an ongoing concern in many countries that highlights the importance of high quality surveillance data) and concerns raised about the methodology utilised for estimating DALYs, this work laid the foundations for the future acknowledgement of NCDs as a true global health threat. More recent data from the GBD group have confirmed that the risk factors defined by the voluntary global NCD targets are large contributors to disease burden [5].

Epidemiology has also done much to explode the myth of NCDs being the preserve of the affluent. As early as in the 1970s, Marmot et al. posited a reversal of the socioeconomic gradient in the association with CVD in the UK, demonstrating that the heart disease mortality rate ratio among groups of higher *v*. lower socioeconomic status changed from 1.2 to 0.9 between 1949–1953 and 1970–1972 [6]. While it is difficult to determine the extent to which a similar reversal has occurred or is occurring in LMIC, there is incontrovertible evidence of increasingly adverse NCD risk factor profiles and high NCD prevalence among poorer communities within such countries. There is also strong evidence of accompanying disadvantage in receipt of appropriate treatments. For example, a recent cross-sectional study involving 17 countries showed that in both upper and lower middle-income nations, the prevalence of hypertension was similar in urban and rural populations [7]. While the prevalence of hypertension was lower in rural *v*. urban communities in low income countries (mainly sub-Saharan Africa), almost one-third of the rural adult population aged between 35 and 70 years was affected. Awareness, treatment and control of hypertension were substantially lower in rural compared with urban populations in low-middle and low income countries. Other data from this study demonstrate substantially lower use of appropriate secondary prevention drugs among people with established CVD in rural compared to urban populations in developing economies [8].

High quality epidemiological data have been pivotal in establishing the risk factors and likely causal determinants of NCDs, many of which are the focus of the voluntary targets for the ‘25 × 25’ goal. A key difference between infectious disease epidemiology and that for most NCDs relates to the multiple pathways of causality associated with the latter. Such recognition underscored the need for the very large studies to establish often moderate associations with individual risk factors. An early and often cited example of this is the establishment of cigarette smoking as a likely causal risk factor for lung cancer, through the seminal case control and cohort studies conducted by Doll and Hill in the 1950s [9, 10]. More than half a century later, while epidemiological data show us that smoking rates have substantially declined over the last few decades in most developed economies, they demonstrate the opposite is true of many LMIC. In fact, it is estimated that over 70% of tobacco-related deaths will occur in LMIC by the year 2020 [11]. A recent study indicated that if current trends continue, tobacco control targets will not be achieved in many LMIC by 2025, and indeed tobacco use will increase in several [12].

In addition to collection and analyses of observational data, the discipline of epidemiology has provided the gold standard methodology for establishing the role of drugs and devices to prevent and treat diseases. While the first examples of the archetypal modern double-blind, placebo-controlled randomised controlled trial were applied to infectious diseases, NCDs have particularly benefited from an explosion of therapeutic developments, which in turn have been evaluated rigorously in large scale randomised trials to provide reliable evidence about efficacy and safety. This has led to the availability of a large arsenal of safe, efficacious, and in many cases, increasingly affordable treatments. In turn, epidemiological methods can provide estimates about the benefits accrued from the use of therapies at a population level. For example, a number of modelling studies have attributed the decline in coronary heart disease incidence and mortality observed in developed countries over recent decades to a combination in reduced population-level risk (especially as a result of a decline in smoking rates), as well success with treatments, both for the acute management of coronary events as well as long-term secondary prevention [13].

However, despite the availability of treatments of proven safety and efficacy, large evidence-practice gaps exist worldwide. This has led to the development of what might be described as one of the newer branches of epidemiology, namely implementation science. This discipline is essentially concerned with knowledge translation, developing and providing evidence for strategies to increase the adoption and sustainable scale-up of evidence-based healthcare (i.e. going beyond ‘does it work?’). Fundamentally, implementation science is about changing behaviour, with an understanding that people (policy makers, healthcare administrators, healthcare providers, patients, community members) operate and make decisions in complex environments. To achieve affordable and effective healthcare the local social, cultural, physical (institutions, workforce and resources) and political context all need to be evaluated and navigated. How people will respond is generally also informed by their own experiences, values, norms and learning. Implementation science is therefore characterised by the need to extend the traditional boundaries of epidemiology, and for epidemiologists to collaborate widely with professionals from a range of other disciplines including social science, health economics and various strands of health systems research. Implementation science needs to incorporate a range of both quantitative and qualitative research methodologies that might not only address a specific implementation problem, but also further generalisable knowledge about changing behaviour in the healthcare context. Many would argue that given the limited research funding available to address the needs of LMIC, implementation science deserves some priority in terms of most rapidly realising the health benefits of existing medical knowledge.

Like all branches of science, epidemiology continues to evolve. Today, a scientist involved in the practice of epidemiology may introduce her or himself as a ‘risk factor epidemiologist’, ‘environmental epidemiologist’, ‘genetic epidemiologist’, ‘life-course epidemiologist’, ‘clinical trialist’, or ‘implementation scientist’, amongst many other such titles, with or without a particular disease focus. All such flavours of epidemiology are crucial to address the NCD agenda and a useful unifying conceptual approach is to consider epidemiology as the basic science of public health, where causal inference remains the central and fundamental objective. This is regardless of whether epidemiological approaches are being used in the search for new drivers of disease burden or the discovery of effective, affordable and accessible population-based or clinical approaches to combat these conditions. Most importantly, as has been demonstrated in its use in so many of its forms in addressing the enormous global challenge of preventing and controlling NCDs, epidemiology must continue to act as a critical scientific tool for public health action. For example, a recent modelling study suggests that continuation of current trends in risk factor levels will lead to a relative increase in premature CVD mortality globally by 2025, with a decrease only observed in high income countries [14]. These analyses indicate that in many regions of the world, the ‘25 × 25’ goal will not be met even if all risk factor targets are met. Such epidemiological data highlight the enormous challenge ahead and the likelihood of failure without an immediate and comprehensive multisectoral response across the globe.
